# Actual Data on Essential Trace Elements in Parkinson’s Disease

**DOI:** 10.3390/nu17111852

**Published:** 2025-05-29

**Authors:** Cristina Popescu, Constantin Munteanu, Aura Spînu, Ioana Andone, Roxana Bistriceanu, Ruxandra Postoiu, Andreea Suciu, Sebastian Giuvara, Andreea-Iulia Vlădulescu-Trandafir, Sorina Maria Aurelian, Nadina Liana Pop, Vlad Ciobanu, Gelu Onose

**Affiliations:** 1 Faculty of Medicine, University of Medicine and Pharmacy “Carol Davila”, 020021 Bucharest, Romania; cristina_popescu_recuperare@yahoo.com (C.P.); aura_ko@yahoo.com (A.S.); ioanaandone11@yahoo.com (I.A.); roxana.bistriceanu@rez.umfcd.ro (R.B.); postoiu.ruxandra@yahoo.ro (R.P.); andreea_v_spiroiu@yahoo.com (A.S.); sebastian.giuvara@gmail.com (S.G.); andreea-iulia.trandafir@drd.umfcd.ro (A.-I.V.-T.); sorina.aurelian@umfcd.ro (S.M.A.); gelu.onose@umfcd.ro (G.O.); 2 Neuromuscular Rehabilitation Clinic Division, Clinical Emergency Hospital “Bagdasar-Arseni”, 041915 Bucharest, Romania; 3 Department of Biomedical Sciences, Faculty of Medical Bioengineering, University of Medicine and Pharmacy “Grigore T. Popa” Iași, 700454 Iași, Romania; 4 Clinic of Geriatrics, Hospital of Chronic Diseases “Sf. Luca”, 041915 Bucharest, Romania; 5 Department of Physiology, Iuliu Hațieganu University of Medicine and Pharmacy Cluj-Napoca, Clinicilor Street No. 1-3, 400006 Cluj-Napoca, Romania; popnadina@yahoo.com; 6 Computer Science Department, Politehnica University of Bucharest, 060042 Bucharest, Romania; vlad.ciobanu@upb.ro

**Keywords:** Parkinson’s disease, trace elements, neurodegeneration, oxidative stress, zinc, iron, copper, manganese, selenium, metal homeostasis

## Abstract

“*Sola dosis facit venenum*” (Paracelsus). Essential trace elements, crucial for maintaining neuronal function, have their dysregulation increasingly correlated with neurodegenerative disorders, particularly Parkinson’s disease (PD). This systematic review aims to synthesize recent high-quality evidence regarding the involvement of essential trace elements, such as iron, zinc, copper, manganese, and selenium, in the pathogenesis and, consequently, as potential therapeutic targets of PD. A comprehensive literature search was conducted for articles published between 1 January 2023 and 31 December 2024. Out of an initial pool of 1231 identified studies, 63 met the methodological eligibility criteria according to PRISMA (Preferred Reporting Items for Systematic Reviews and Meta-Analyses) guidelines. All potentially eligible interventional and observational studies were initially assessed using the Physiotherapy Evidence Database (PEDro) scale, which is commonly employed for evaluating the internal validity and statistical interpretability of clinical trials and rehabilitation-focused studies. Following the qualitative assessment using the PEDro scale, 18 studies were ultimately selected based on their scientific relevance and methodological rigor. To supplement the PEDro scoring, which is designed primarily for individual trials, we applied the AMSTAR-2 (A MeaSurement Tool to Assess Systematic Reviews) checklist for the evaluation of the included systematic reviews or meta-analyses. The included studies employed a variety of clinical, postmortem, and experimental models to investigate trace-element concentrations and their mechanistic roles in PD. The findings revealed consistent patterns of iron accumulation in the substantia nigra, zinc’s bidirectional effects on oxidative stress and autophagy, copper-induced α-synuclein aggregation, and the neuroprotective role of selenium via antioxidant pathways. Manganese was associated with mitochondrial dysfunction and neuroinflammation. Essential trace-element disturbances contribute to PD pathology through interconnected mechanisms involving redox imbalance, protein misfolding, and impaired cellular homeostasis. These elements may serve as both biomarkers and potential therapeutic tools, warranting further investigation into personalized metal-based interventions for PD.

## 1. Introduction

Parkinson’s disease (PD) is one of the most prevalent chronic progressive neurodegenerative disorders worldwide, affecting over 10 million individuals, and is characterized clinically by gradual motor dysfunction such as resting tremor, rigidity, bradykinesia, and postural instability, and pathologically by the degeneration of dopaminergic neurons in the substantia nigra pars compacta (SNpc), and the consequent depletion of dopamine in the nigrostriatal pathway [1,2]. In addition to motor manifestations, PD encompasses a broad spectrum of non-motor symptoms, including cognitive impairment, mood disorders, sleep disturbances, anosmia, and autonomic dysfunction, many of which may precede the motor deficits by several years [3]. As it is well established for chronic diseases in general, their initiation, progression, and eventual prognosis are influenced by a complex interplay of environmental, biological [4], behavioral, and physical exercise [5], socioeconomic factors, as well as by broader public health issues, or living standards [6,7].

**Figure 1:**
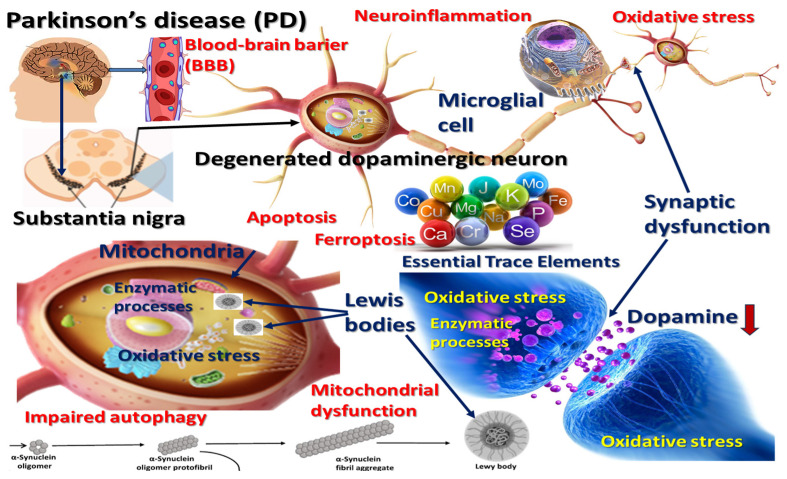
The role of essential trace elements in Parkinson’s disease pathophysiology: mechanisms involving oxidative stress, mitochondrial dysfunction, neuroinflammation, and impaired autophagy. Disruption of the blood–brain barrier (BBB) facilitates trace-element dyshomeostasis and neurotoxicant entry. Essential trace elements—including iron (Fe), copper (Cu), zinc (Zn), manganese (Mn), selenium (Se), chromium (Cr), molybdenum (Mo), iodine (I), and others—are depicted, emphasizing their dual potential to support normal enzymatic activities or exacerbate neurodegeneration when dysregulated.

While the etiopathogenesis of PD is multifactorial—incorporating genetic predisposition, environmental toxins, and aging—the disruption of trace elements’ homeostasis has emerged as a compelling, yet underexplored, contributor to disease development and progression [8,9,10]. Essential trace elements such as iron (Fe), copper (Cu), zinc (Zn), manganese (Mn), and selenium (Se) play pivotal roles in maintaining neuronal function, acting as cofactors in enzymatic processes, redox signaling, and mitochondrial respiration. However, both a deficiency and an excess of these elements may induce cellular dysfunction through mechanisms such as oxidative stress, ferroptosis [11], impaired autophagy, and abnormal protein aggregation, particularly of α-synuclein (see Figure 1) [12,13].

The brain’s vulnerability to oxidative stress positions trace elements at the center of neurodegenerative disease mechanisms [14]. When dysregulated, they contribute to the overproduction of reactive oxygen species (ROS) and reactive nitrogen species (RNS) [15], initiating a cascade of oxidative damage to lipids, carbohydrates, proteins, nucleic acids, and organellar membranes [16].

Environmental [17] and nutritional factors are increasingly recognized as significant contributors to PD risk and progression [18]. Epidemiological evidence supports an association between chronic exposure to environmental toxins, such as pesticides (e.g., paraquat, rotenone), herbicides, and industrial solvents, and an elevated risk of developing PD [19,20]. These agents often target mitochondrial respiratory complexes or promote α-synuclein aggregation [21]. Exposure to air pollutants, particularly fine particulate matter and nitrogen dioxide (NO_2_), has been linked to systemic inflammation and neurotoxicity [22], potentially affecting the olfactory system and the gut–brain axis [23,24]. Elevated brain levels of iron, copper, manganese, and zinc have been detected in PD patients, contributing to oxidative stress, α-synuclein misfolding, and mitochondrial dysfunction [25]. Conversely, deficiencies in protective micronutrients, such as selenium, magnesium, and specific vitamins, may compromise neuronal resilience by impairing antioxidant defense systems [26].

This systematic review aims to provide a comprehensive synthesis of the most recent evidence concerning the role of essential trace elements in the pathogenesis, diagnosis, and potential therapeutic modulation of PD. It evaluates alterations in trace-element concentrations across various biological matrices—including serum, cerebrospinal fluid (CSF), hair, and brain tissues—and examines their mechanistic impact on neuronal redox homeostasis. Moreover, the review explores the contribution of trace-element dysregulation to key molecular hallmarks of PD pathology, such as oxidative stress, mitochondrial dysfunction, α-synuclein aggregation, neuroinflammatory processes, and ferroptosis.

## 2. Materials and Methods

This systematic literature review was conducted under the PRISMA (Preferred Reporting Items for Systematic Reviews and Meta-Analyses) guidelines [27] to critically analyze and synthesize recent evidence regarding the role of essential trace elements in the pathogenesis of PD. Furthermore, it sought to evaluate the potential of trace element-based preventive or therapeutic interventions designed to counteract or delay PD onset and mitigate its subsequent progression.

The primary objective of this review was to identify, contextualize, and integrate the most up-to-date evidence linking essential trace elements—specifically zinc, iron, copper, manganese, selenium, and others—to the initiation, evolution, and potential treatment strategies of PD. Special emphasis was placed on studies addressing both empirical clinical findings and underlying mechanistic pathways, including their relevance for biomarker discovery and therapeutic modulation.

The literature search encompassed two consecutive calendar years, covering publications from 1 January 2023 to 31 December 2024. Systematic searches were performed across six major academic databases—PubMed, PMC, Web of Science, Scopus, and PEDro—to ensure comprehensive coverage of high-impact and ISI-indexed journal publications. The search strategy employed both controlled vocabulary terms (MeSH) and tailored combinations of free-text keywords, including but not limited to: “Parkinson’s Disease”, “trace elements”, “zinc”, “iron”, “copper”, “manganese”, “selenium”, “iodine”, “chromium”, “nickel”, “vanadium”, “lithium”, “fluoride”, “molybdenum”, and “strontium”. Boolean operators (AND, OR) and truncation techniques were utilized to maximize the breadth and specificity of the search while maintaining relevance to the research question. In addition to database searches, supplementary bibliographic resources, mainly from 2025, were incorporated, including relevant studies identified through manual searches of reference, thereby enhancing the comprehensiveness of the evidence base.

**Figure 2:**
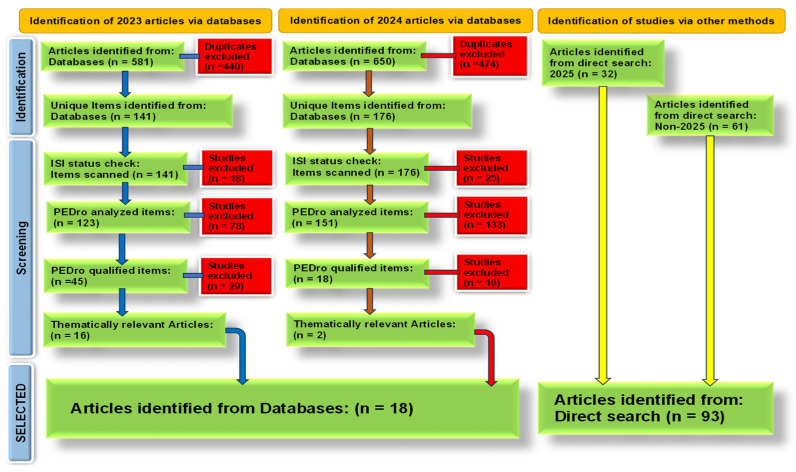
PRISMA flow diagram adapted to our study.

The inclusion criteria mandated that studies be original research articles published in English, focusing explicitly on the role of essential trace elements in PD or at least neurodegenerative disorders in general. Eligible studies included those examining mechanisms of action, clinical significance, and analytical quantification of trace elements in human subjects or experimental models (in vivo or in vitro) (see Figure 2).

Due to the significant methodological heterogeneity observed across the included studies, ranging from clinical case–control analyses to mechanistic laboratory investigations, a quantitative meta-analysis was deemed unfeasible. In lieu of a meta-analysis, a qualitative synthesis was performed, systematically organized according to specific trace elements and mechanistic themes.

Based on the AMSTAR-2 structured quality assessment, methodological standards regarding research questions and inclusion criteria included the PICO components (Population: individuals with Parkinson’s Disease; Intervention: exposure or modulation of essential trace elements; Comparator: healthy controls or baseline; Outcome: involvement in pathogenesis and therapeutic potential). In conclusion, the manuscript achieves a commendable level of methodological rigor and comprehensiveness, particularly in literature searching and qualitative synthesis.

The narrative synthesis integrates convergent lines of evidence, elucidating both the synergistic and antagonistic effects of trace elements on PD pathophysiology. Furthermore, it offered broader insights by identifying mechanistic overlaps between PD and Alzheimer’s disease (AD), thus contributing to a more comprehensive understanding of trace-element dysregulation in neurodegenerative processes.

## 3. Results

The systematic review is based on 18 relevant high-quality articles, 16 from 2023 and 2 from 2024, selected from a pool of 1231 identified articles, addressing the role of essential trace elements in PD. The selected studies covered a spectrum of elements, focusing on zinc, iron, copper, selenium, and manganese. Several articles reported elevated iron accumulation in the substantia nigra and its association with oxidative stress, mitochondrial dysfunction, and dopaminergic neuronal loss. Zinc was highlighted for its dual role, functioning as both a neuroprotective and neurotoxic agent depending on concentration and cellular context, often mediated through metal transporters and autophagy pathways. Copper dysregulation was consistently linked to α-synuclein aggregation and oxidative stress, while selenium was examined in the context of antioxidant defense, especially glutathione peroxidase activity. Manganese exposure was implicated in mitochondrial impairment and neuroinflammation. Overall, the reviewed articles agree that imbalances in trace-element homeostasis contribute to PD pathophysiology through redox-mediated mechanisms, impaired protein clearance, and neuroinflammatory responses.

### 3.1. Trace Elements: Biological Functions and Brain Homeostasis

**Figure 3:**
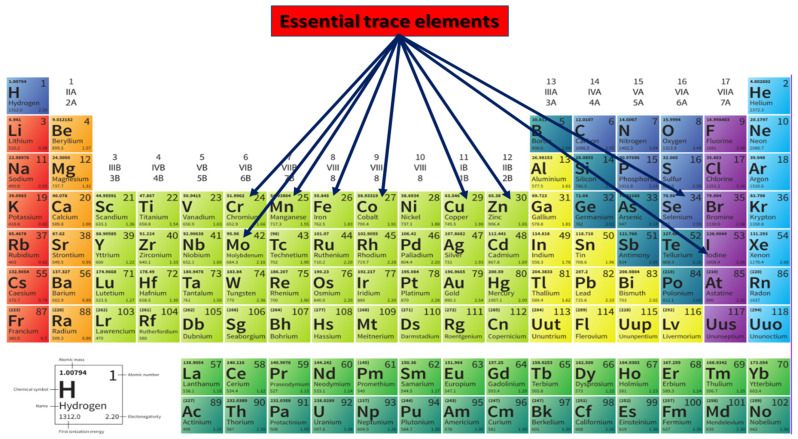
Essential trace elements.

Besides major elements, such as sodium (Na) [28] or calcium (Ca) [29], trace elements are broadly categorized into two principal groups based on their biological necessity: essential and non-essential [30]. *Essential trace elements* (see Figure 3) are indispensable for normal cellular and systemic functions [31]. Their absence or deficiency leads to specific physiological impairments, possibly reversible upon repletion. This group includes iron (Fe), copper (Cu), zinc (Zn), manganese (Mn), selenium (Se), iodine (I), cobalt (Co), chromium (Cr), and molybdenum (Mo). These elements are typically incorporated into metalloenzymes, metalloproteins, and structural biomolecules that govern redox homeostasis, mitochondrial respiration, neurotransmitter synthesis, and immune function [32]. For example, iron is required for heme-containing cytochromes and dopamine synthesis [33]; copper and zinc are cofactors in superoxide dismutase (SOD1) [34]; manganese supports mitochondrial antioxidant defenses via Mn-SOD [35] and selenium is essential for glutathione peroxidase (GPX) activity [36].

In contrast, *non-essential trace elements* are not required for biological processes, nor for which a definitive physiological role has been established. Some non-essential elements, such as lead (Pb) [37], mercury (Hg), arsenic (As), cadmium (Cd), and aluminum (Al), are considered neurotoxic and can accumulate, including in neural tissues, where they interfere with essential metal homeostasis, disrupt cellular signaling, and promote neurodegeneration. Although they are not central to normal cellular function, emerging research suggests that even some non-essential elements may exert subtle biological effects, whether beneficial or harmful, depending on their concentration, chemical speciation, and environmental context [38].

A key regulator of trace-element homeostasis in the central nervous system (CNS) is the blood–brain barrier (BBB)—a highly selective, semipermeable interface, consisting of neurovascular units, formed mainly by brain microvascular endothelial cells, astrocytic end-feet, and pericytes [39]. The BBB restricts the passive diffusion of most substances and facilitates the regulated transport of essential nutrients and metals via specialized transporter systems [40]. The BBB acts as a gatekeeper and a dynamic regulator, also for trace elements [41].

### 3.2. Trace-Element Levels Altered in Parkinson’s Disease

Iron (Fe) is a critical trace element in the CNS, where it plays essential roles in cellular respiration, neurotransmitter synthesis, and myelin formation [42]. Iron is particularly important in dopaminergic neurons due to its involvement in the enzymatic conversion of tyrosine to L-DOPA via tyrosine hydroxylase, a rate-limiting step in dopamine biosynthesis [43]. Furthermore, iron serves as a cofactor for numerous mitochondrial enzymes, particularly those within the electron transport chain (ETC), including complexes I, II, and III, where it is necessary for efficient ATP generation [44].

Recent mechanistic insights also involve iron in the intimacy of ferroptosis, a non-apoptotic form of cell death driven by iron-dependent lipid peroxidation [11]. Ferroptosis has gained prominence in PD research, as it aligns with the observed features of iron accumulation, glutathione depletion, and reduced glutathione peroxidase 4 (GPX4) activity in affected neurons. Inhibitors of ferroptosis, such as deferoxamine (DFO), deuterated PUFAs, and Ferrostatin-1 (Fer-1), have shown neuroprotective effects in PD models [45], further substantiating the role of iron in this degenerative pathway [1,37].

Copper (Cu) is an essential trace element with multifaceted roles in the CNS, where it contributes to enzymatic catalysis, neurotransmitter synthesis, redox balance, and mitochondrial respiration [46]. As a cofactor for enzymes such as cytochrome c oxidase (complex IV of the mitochondrial ETC), dopamine β-hydroxylase, and Cu/Zn-superoxide dismutase (SOD1), copper is indispensable for oxidative phosphorylation, catecholamine biosynthesis, and antioxidant defense [47].

A key mechanism linking copper to PD pathology involves its direct interaction with α-synuclein, the principal component of Lewy bodies. Copper binds to α-synuclein with high affinity at specific histidine and methionine residues, particularly at His50, a region commonly mutated in familial PD. This binding promotes a conformational shift in α-synuclein from a disordered to a β-sheet-rich structure, accelerating its oligomerization and fibril formation [48]. Furthermore, copper-bound α-synuclein acquires redox activity, producing hydrogen peroxide and superoxide radicals, exacerbating oxidative stress and propagating neuronal damage. In vitro studies demonstrate that copper accelerates the chemical nucleation phase of α-synuclein fibrillization and increases its cytotoxicity [1,47].

Recent epidemiological data from large-scale studies, including the National Health and Nutrition Examination Survey (NHANES) cohorts, suggest that dietary copper intake above a defined threshold (>1.96 mg/day) may be inversely associated with PD risk, particularly in specific subpopulations such as middle-aged adults, women, and individuals with higher education levels [47]. These findings imply that optimal copper levels may be neuroprotective, whereas dysregulation, either in deficiency or overload, can promote neurodegenerative processes [49].

Zinc (Zn) is a highly abundant essential trace element in the human brain, second only to iron in total concentration, and plays multifaceted roles in maintaining neural function and structural integrity [50]. It is a critical cofactor for over 300 enzymes involved in DNA replication, antioxidant defense, and neurotransmission [51,52]. In the CNS, zinc is concentrated in synaptic vesicles of glutamatergic neurons, particularly in regions such as the hippocampus, amygdala, and neocortex. It modulates synaptic activity through its influence on ionotropic receptors (NMDA, AMPA) and participates in neurogenesis, plasticity, and repair [53]. Furthermore, zinc contributes to antioxidant defense as a structural component of Cu/Zn-superoxide dismutase (SOD1), thus playing a protective role against oxidative damage [51].

In PD, alterations in zinc homeostasis have been increasingly recognized as contributing factors to the disease pathogenesis [53]. Postmortem studies of PD brains have shown dysregulated zinc concentrations in several affected regions, including the substantia nigra, where both accumulation and depletion have been reported [54]. Elevated intracellular zinc may result from increased synaptic release or impaired efflux through zinc transporters. Under these conditions, free Zn^2+^ acts as a neurotoxin by promoting oxidative stress, mitochondrial dysfunction, and protein aggregation [55]. Conversely, zinc deficiency may compromise neuronal antioxidant capacity and weaken synaptic signaling, highlighting the complexity of zinc’s role in PD [56].

Zinc has been shown to regulate autophagy, a key cellular mechanism for clearing damaged proteins and organelles. Dysregulated zinc levels impair lysosomal acidification and autophagic flux, promoting the accumulation of α-synuclein aggregates—a hallmark of PD pathology. Moreover, zinc-induced inhibition of vacuolar-type H^+^-ATPase disrupts endolysosomal integrity, exacerbating proteostasis failure in dopaminergic neurons [57].

Emerging evidence also points to zinc involvement in ferroptosis, an iron-dependent, non-apoptotic cell death driven by lipid peroxidation. Although traditionally associated with iron, zinc modulates ferroptosis through its interaction with antioxidant systems such as GPX4 and system Xc^−^, which regulates cystine uptake and glutathione synthesis. Zinc deficiency impairs GPX4 activity indirectly, rendering neurons more susceptible to ferroptotic stress, while zinc overload promotes lipid peroxidation and ferroptosis-like phenotypes in dopaminergic cell models [56,58].

Recent research has deepened our understanding of zinc homeostasis in PD, particularly emphasizing the role of zinc transporter proteins as the Zrt-Irt-like protein (ZIP) and Zn transporter (ZnT) families [59]. These transporters regulate intracellular zinc distribution, and their dysregulation may exacerbate neurodegeneration. ZnT1, ZnT3, and ZnT10, for instance, modulate zinc efflux into the extracellular matrix or synaptic vesicles, while ZIP7 and ZIP14 facilitate zinc influx. Dysfunction of these transporters can perturb the redox balance and promote ferroptosis. Moreover, ZIP7-mediated zinc efflux from the endoplasmic reticulum has been implicated in activating ferroptotic pathways through modulation of endoplasmic reticulum (ER) stress and homocysteine-inducible ER protein with ubiquitin-like domain 1 (HERPUD1) signaling [60]. These insights elaborate on the dual role of zinc in neuroprotection and neurotoxicity and provide a rationale for targeting specific transporter isoforms as therapeutic strategies to mitigate PD progression [52].

Manganese (Mn) is an essential trace element required for normal CNS function, playing key roles as a cofactor in several enzymes involved in antioxidant defense, neurotransmitter synthesis, and energy metabolism [35]. Notably, manganese is vital for the activity of mitochondrial SOD (Mn-SOD), arginase, and glutamine synthetase, which are critical for mitigating oxidative stress and supporting neuronal metabolism [61]. However, manganese exhibits a narrow therapeutic window, and excessive accumulation in the brain, particularly within the basal ganglia, can lead to neurotoxicity. This pathological state, known as *manganism*, shares clinical and neuropathological features with PD, including motor rigidity, bradykinesia, and postural instability, but differs in its lack of response to levodopa and absence of Lewy bodies [37].

Beyond mitochondrial impairment, manganese promotes the aggregation of α-synuclein. Experimental studies demonstrate that Mn exposure upregulates α-synuclein expression, predisposes to misfolding, and enhances exosome cell transmission. This process is partly mediated by oxidative stress and inflammatory signaling, suggesting that Mn contributes to both the initiation and propagation of α-synuclein pathology [62]. Additionally, manganese triggers neuroinflammatory responses by activating microglia and inducing the nucleotide-binding oligomerization domain-like receptor family pyrin domain containing 3 (NLRP3) inflammasome, releasing pro-inflammatory cytokines such as IL-1β and TNF-α, which amplify neuronal damage [63].

Environmental and occupational exposure to manganese constitutes a significant risk factor for neurotoxicity. Populations at risk include welders, miners, battery factory workers, and agricultural laborers, where the inhalation of Mn-containing particulates leads to its entry into the brain via olfactory and trigeminal pathways, bypassing the BBB [64]. Chronic exposure is associated with progressive motor and cognitive decline and has been epidemiologically linked to an increased incidence of Parkinsonian syndromes. Environmental Mn exposure has also been detected in populations with high Mn levels in drinking water or soil, suggesting a broader relevance to public health [37].

Selenium (Se) is an essential trace element in maintaining redox homeostasis, neuroprotection, and cellular antioxidant defense mechanisms. In the CNS, selenium exerts its biological effects predominantly by incorporating into a family of selenium-containing proteins known as selenoproteins. Among these, GPX—particularly GPX1 and GPX4—and thioredoxin reductase (TrxR) are of critical importance in protecting neurons against oxidative damage [65].

Several postmortem and experimental studies have indicated that selenoprotein expression and activity are reduced in PD, contributing to an environment of heightened oxidative stress and increased vulnerability to neuronal death. Moreover, selenium deficiency has been shown to exacerbate mitochondrial dysfunction, decrease glutathione synthesis, and facilitate the aggregation of misfolded proteins, including α-synuclein [66].

Recent advances in selenium-based nanotechnology have extended its therapeutic potential. For instance, selenium–human serum albumin nanoparticles (Se/HSA-NPs) have been engineered for oral delivery and shown to cross the BBB efficiently. These nanoparticles target the substantia nigra, enhance GPX4 expression, and inhibit ferroptosis, thereby preserving mitochondrial integrity and motor function in PD animal models [67].

An expanding body of evidence indicates that iodine (I) and several lesser-studied ultra-trace elements—including chromium (Cr), nickel (Ni), vanadium (V), lithium (Li), fluoride (F), strontium (Sr), and molybdenum (Mo)—may also contribute to neurodegenerative processes. These elements appear to exert their effects primarily through mechanisms involving oxidative stress, mitochondrial dysfunction, neuroinflammatory responses, and epigenetic modifications. Although the available data on these less extensively investigated elements remain comparatively limited and often heterogeneous in quality, emerging findings increasingly support their consideration in the broader context of trace element-related neurotoxicity and their potential role as modulators of PD pathophysiology [68].

### 3.3. Focused Synthesis of Molecular and Cellular Mechanisms

Oxidative stress is a central pathogenic mechanism in PD, resulting from an imbalance between ROS production and the antioxidant defense systems’ capacity to neutralize them [69]. The brain is particularly susceptible to oxidative insults due to its high oxygen consumption, abundant polyunsaturated fatty acids, and relatively low antioxidant reserves [70]. Dopaminergic neurons in the substantia nigra are even more vulnerable due to dopamine metabolism itself being a source of ROS and reactive quinones. Within this context, essential trace elements such as iron (Fe), copper (Cu), and manganese (Mn) play pivotal roles in ROS generation and oxidative injury via their redox-active properties [71].

Iron, particularly in its ferrous form (Fe^2+^), is a major contributor to free radical production through the Fenton reaction, in which Fe^2+^ reacts with hydrogen peroxide (H_2_O_2_) to generate hydroxyl radicals (•OH)—among the most reactive and damaging species known. These hydroxyl radicals initiate lipid peroxidation, protein oxidation, and DNA strand breaks, ultimately resulting in mitochondrial damage and apoptotic or ferroptotic cell death [72]. In addition, the Haber–Weiss reaction provides another pathway for ROS amplification, where superoxide (O_2_•^−^) reduces ferric iron (Fe^3+^) back to Fe^2+^, maintaining the Fenton cycle: O_2_•^−^ + Fe^3+^ → O_2_ + Fe^2+^. This cyclical, vicious regeneration of Fe^2+^ perpetuates oxidative injury and is a key feature of PD pathophysiology, as postmortem analyses consistently show elevated iron levels in the substantia nigra of PD patients [73].

Copper similarly participates in ROS generation via redox cycling between monovalent Cu⁺ and bivalent Cu^2+^. Although copper is essential for the activity of antioxidant enzymes such as SOD 1 (Cu/Zn-SOD), excessive free copper catalyzes Fenton-like reactions analogous to iron, contributing to oxidative stress [74]. Additionally, copper has a high affinity for α-synuclein and accelerates its misfolding and aggregation in vitro, thus enhancing the production of hydrogen peroxide and other ROS in the process. In PD, alterations in copper homeostasis have been observed, with both regional accumulations and systemic deficiencies reported, suggesting that copper imbalance contributes to redox disruption at multiple levels [75].

Manganese, while less directly involved in classical Fenton chemistry due to its less reactive redox potential, contributes to oxidative stress through its impact on mitochondrial integrity and antioxidant systems. At physiological levels, Mn is essential for mitochondrial SOD (Mn-SOD), which detoxifies superoxide radicals in the mitochondrial matrix [35]. However, in conditions of manganese overload, excess Mn interferes with mitochondrial ETC complexes, leading to electron leakage and the overproduction of superoxide and hydrogen peroxide. Moreover, Mn disrupts calcium homeostasis and promotes excitotoxicity, further compounding oxidative damage. Excessive Mn also induces endoplasmic reticulum stress and activates the unfolded protein response, both linked to ROS production and cell death [76].

Neuroinflammation is a prominent and sustained pathological feature of PD, characterized by the activation of glial cells, particularly microglia, and the chronic release of pro-inflammatory cytokines and chemokines. Microglial activation contributes to the progression of neurodegeneration by amplifying oxidative stress, promoting synaptic dysfunction, and inducing neuronal apoptosis [77]. Among the key molecular players orchestrating this inflammatory response is the NLRP3 inflammasome, a cytosolic multiprotein complex that acts as a molecular sensor for cellular danger signals, including oxidative stress, mitochondrial dysfunction, and misfolded proteins such as α-synuclein. Once activated, the NLRP3 inflammasome facilitates the cleavage of pro-caspase-1 into its active form, leading to the maturation and release of interleukin-1β (IL-1β) and interleukin-18 (IL-18), which perpetuate neuroinflammatory cascades [78].

The misfolding and aggregation of α-synuclein is a defining pathological hallmark of PD, with oligomeric and fibrillar forms of this protein comprising the major constituents of Lewy bodies and neurites observed in affected brain regions. While α-synuclein is a physiologically soluble presynaptic protein involved in synaptic vesicle trafficking, neurotransmitter release, and membrane remodeling, its structural flexibility and intrinsic disorder render it highly susceptible to conformational alterations under pathogenic conditions. Among the most influential modulators of α-synuclein aggregation are trace metals—particularly iron (Fe), copper (Cu), zinc (Zn), and manganese (Mn)—which interact directly with the protein and promote its pathological transformation into β-sheet-rich aggregates [79,80].

Copper exhibits a strong affinity for α-synuclein and binds primarily to histidine residues in its *N*-terminal region, notably His50, also a mutation site in familial PD. The binding of Cu^2+^ induces a conformational transition in α-synuclein, promoting its compaction and favoring oligomerization and fibril nucleation. Furthermore, copper-bound α-synuclein becomes redox-active, catalyzing the production of ROS, particularly hydrogen peroxide and superoxide radicals. This redox cycling exacerbates oxidative stress and leads to oxidative modifications of α-synuclein itself, including tyrosine nitration and dityrosine cross-linking, stabilizing toxic oligomeric species resistant to degradation. Such changes also impair the autophagic and proteasomal clearance of α-synuclein, contributing to its accumulation in neurons [81].

Iron similarly facilitates α-synuclein aggregation through both direct and indirect mechanisms. α-synuclein contains a putative iron-responsive element (IRE) in its 5′ untranslated region, which allows its expression to be upregulated by iron. Elevated intracellular Fe^3+^ binds to the *C*-terminal region of α-synuclein, inducing structural rearrangements and promoting the formation of insoluble aggregates. Iron also accelerates α-synuclein fibrillization in vitro and enhances the stability of preformed fibrils. In dopaminergic neurons, excess iron catalyzes oxidative stress via Fenton chemistry, indirectly promoting protein oxidation, lipid peroxidation, and neuronal membrane damage, all of which contribute to a permissive environment for α-synuclein aggregation. Moreover, iron-induced oxidative modifications may facilitate the formation of toxic α-synuclein protofibrils that disrupt mitochondrial membranes and impair cellular homeostasis [82].

Zinc, although less redox-active than iron or copper, modulates α-synuclein aggregation by altering its conformational dynamics. Zinc binds to acidic residues within the *C*-terminal domain of α-synuclein and induces its partial folding, accelerating the transition from monomers to oligomers. In vitro studies demonstrate that zinc promotes α-synuclein fibrillation and stabilizes intermediate species that are particularly neurotoxic. Zinc also interacts with synaptic vesicle pools and can modulate α-synuclein’s physiological role in vesicle recycling. The dysregulation of zinc transporters (ZnTs and ZIPs) in PD models has been associated with increased synaptic zinc levels, creating a microenvironment conducive to α-synuclein aggregation and synaptic dysfunction [83].

Manganese, while not directly binding α-synuclein with high affinity, exerts its effects by upregulating α-synuclein expression and enhancing its aggregation indirectly. Mn exposure has been shown to increase α-synuclein mRNA and protein levels via oxidative stress and mitochondrial dysfunction. Additionally, manganese promotes the release of α-synuclein into the extracellular space and enhances its transmission between neurons through exosomal and prion-like mechanisms [62]. Thus, manganese not only contributes to α-synuclein accumulation but may also facilitate its intercellular propagation, a process increasingly recognized as central to PD progression [84].

### 3.4. Trace Elements as Biomarkers in PD

Assessing trace-element concentrations in biological fluids such as serum and cerebrospinal fluid (CSF) has emerged as a promising avenue for identifying potential biomarkers in PD (see Table 1). These biomarkers reflect disease presence, progression, and pathophysiological mechanisms such as oxidative stress, mitochondrial dysfunction, or protein aggregation. However, while numerous studies have reported alterations in trace-metal levels in PD patients, results have often been inconsistent, highlighting the complexity of interpreting these measurements in both peripheral and central compartments [10,85].

Serum studies have reported significant deviations in the concentrations of essential trace elements such as iron (Fe), copper (Cu), zinc (Zn), manganese (Mn), and selenium (Se) in PD patients compared to healthy controls. For example, several investigations have documented elevated serum Fe and Cu levels, potentially reflecting systemic inflammation and redox imbalance. Conversely, other studies have found decreased serum Cu and Zn levels, potentially indicating impaired absorption or increased tissue sequestration. Selenium levels in serum are frequently reported as reduced in PD patients, consistent with diminished antioxidant defense mediated by selenoproteins such as GPX. However, these findings vary by geographic region, age, diet, disease stage, and analytical methods employed [31,41].

CSF concentrations may not always mirror serum levels due to the regulatory role of the blood–brain barrier (BBB) and active transport mechanisms that control the movement of metals between systemic circulation and the CNS. For instance, the transferrin and ceruloplasmin-mediated transport of iron and copper is tightly regulated at the BBB, and disruptions in these systems, common in PD, may contribute to central–peripheral disparities in trace-element profiles [57].

Hair, nail, and urine analyses have garnered increasing interest as non-invasive tools for assessing trace-element exposure and potential biomarker discovery in PD. Unlike serum or CSF, which primarily reflect short-term or dynamic changes in metal concentrations, keratinized tissues such as hair and nails and excretory fluids like urine provide a longer-term integrative measure of trace-element accumulation, particularly for elements with chronic exposure patterns. These matrices are especially valuable for population-based studies and environmental or occupational health surveillance, as they enable minimally invasive and repeatable sampling [86,87].

Urine analysis represents a practical and widely employed method for assessing both recent and cumulative exposure to trace elements and their excreted metabolites. In PD cohorts, manganese and iron urine levels have been variably reported as elevated, especially in populations with known environmental or occupational exposure (e.g., welders, miners). Urinary selenium concentrations are often reduced in PD patients, reflecting impaired selenium metabolism or utilization. However, urinary concentrations can be influenced by hydration status, renal function, and circadian rhythms, necessitating normalization to creatinine or other correction factors for accurate interpretation [88].

**Table 1:** Concentrations of trace elements in Parkinson’s disease (PD).

Trace Element	Biological Tissue	Concentration in PD Patients	Concentration in Controls	Scientific Interpretation	Reference
Zinc (Zn)	Human body	Lower levels in serum, plasma, and CSF	2–3 g	The zinc content in the CSF is significantly increased in the brains of PD patients.	[52,59]
Zinc (Zn)	Serum	964 ± 360 µg/L	1026 ± 323 µg/L	Slight, non-significant decrease in serum Zn; possible systemic redistribution	[31]
Zinc (Zn)	Hair	619.58 ± 262.50 µg/g	459.30 ± 187.10 µg/g	Systemic redistribution; oxidative shift in hair Zn levels	[87]
Zinc (Zn)	Adult human brain	-	10 µg/g	Post-mortem studies revealed excessive zinc depositions in the substantia nigra	[53,55]
Iron (Fe)	Serum	949 ± 325 µg/L	1130 ± 428 µg/L	Iron dysregulation is associated with oxidative stress and mitochondrial injury	[31]
Iron (Fe)	Hair	50.98 ± 11.84 µg/g	72.6 ± 44.8 µg/g	Reduced Fe levels linked to neurodegenerative stress	[87]
Copper (Cu)	Serum	1116 ± 258 µg/L	1152 ± 282 µg/L	Minor systemic fluctuation; no clear trend; localized brain Cu accumulation	[47]
Manganese (Mn)	Serum	1.27 ± 0.81 µg/L	2.02 ± 0.50 µg/L	Substantial serum Mn reduction correlating with environmental and mitochondrial impact	[31]
Selenium (Se)	Serum	73.0 ± 18.3 µg/L	109.8 ± 16.9 µg/L	Se depletion associated with impaired antioxidant defense in PD	[66]

Neuroimaging has emerged as a powerful, non-invasive approach for in vivo assessment of metal deposition in the brain, offering valuable insights into the spatial distribution and pathological significance of trace-element dysregulation in PD. Among the various imaging modalities, magnetic resonance imaging (MRI) and positron emission tomography (PET) are particularly instrumental in detecting and quantifying abnormal accumulations of metals such as iron (Fe), copper (Cu), manganese (Mn), and zinc (Zn) [8,89].

Despite their promise, non-invasive biomarkers face several limitations. First, external contamination from environmental sources (e.g., shampoos, soil, airborne particles) can confound hair and nail analyses, requiring meticulous sample preparation and decontamination protocols. Second, inter-individual variability in trace-element absorption, excretion, and incorporation—driven by genetics, diet, comorbidities, and medication use—may obscure disease-specific patterns. Third, unlike CSF, peripheral matrices do not necessarily reflect CNS metal levels or bioavailability, although they can serve as surrogates for systemic dysregulation [90].

### 3.5. Prophylactic and Therapeutic Implications

Dietary supplementation and nutritional interventions, including drinking mineral waters [91,92] or using various balneary resources [93], targeting essential trace elements offers a complementary, non-invasive, and potentially neuroprotective strategy in the management of PD, considering that trace elements most frequently involved in PD (Se, Zn, and Mg), are all essential for enzymatic function, redox balance, and cellular integrity [94]. This prophylactic approach is grounded in the growing body of evidence linking trace-element deficiencies and imbalances to key pathogenic mechanisms in PD, including oxidative stress, mitochondrial dysfunction, α-synuclein aggregation, and neuroinflammation. Nutritional supplementation aims to restore optimal levels of these micronutrients, thereby enhancing endogenous neuroprotective systems and mitigating the deleterious effects of disease-related oxidative and inflammatory processes [95,96].

Chelation therapy represents a targeted therapeutic approach aimed at mitigating the neurotoxic effects of excess trace-metal accumulation in neurodegenerative diseases such as PD. The rationale for chelation in PD arises from the well-established role of redox-active metals—particularly Fe and Cu—in promoting oxidative stress, α-synuclein aggregation, and neuronal loss. Chelating agents are small molecules capable of binding metal ions with high affinity, thereby facilitating their removal, redistribution, or inactivation and reducing their pro-oxidant activity. Several chelators, such as deferoxamine, deferasirox, and deferiprone, have been investigated for their efficacy in modifying disease progression in PD, with varying degrees of clinical success and safety profiles [97].

Metal-targeted drug delivery systems have emerged as an innovative and precision-oriented therapeutic strategy in PD, particularly in addressing the complex challenges posed by trace-element dyshomeostasis, oxidative stress, and neurodegeneration. Traditional approaches such as oral chelation or dietary supplementation often suffer from limited selectivity, poor blood–brain barrier (BBB) penetration, and systemic toxicity. In contrast, nanoparticle-based platforms and intelligent chemical technologies (ICT)-based probes offer enhanced targeting, controlled release, and real-time monitoring capabilities, thus maximizing therapeutic efficacy while minimizing off-target effects [98,99].

Intelligent chemical technologies (ICT)-based probes, also known as activatable or environment-sensitive probes, represent a highly refined strategy for simultaneously diagnosing and modulating metal-associated neuropathology. These probes are designed to respond to specific stimuli in the brain microenvironment, such as redox potential, pH shifts, metal ion concentrations, or enzymatic activity. Upon activation, they can release therapeutic payloads (e.g., chelators, antioxidants) or emit imaging signals for real-time tracking. For example, metal-responsive fluorescent probes capable of binding Fe^2+^, Cu^+^, or Zn^2+^ have been developed for in vivo imaging using near-infrared (NIR) or MRI modalities. These tools enable dynamic monitoring of trace-metal distribution and accumulation in the substantia nigra, facilitating early diagnosis and treatment stratification [100].

Nanoparticles (NPs) have been extensively experimentally investigated for their ability to deliver metal-modulating agents directly to affected neural tissues [99]. These nanoscale carriers can be engineered from various biocompatible materials—such as lipids, polymers (e.g., poly(lactic-co-glycolic) acid (PLGA), metals (e.g., gold, selenium), and inorganic oxides—and functionalized with surface ligands to cross the BBB and target specific cellular populations, such as dopaminergic neurons or activated microglia. For instance, selenium nanoparticles (SeNPs) have demonstrated significant neuroprotective effects in PD models by enhancing selenoenzyme activity (e.g., GPX4), suppressing ferroptosis, and scavenging ROS. The functionalization of SeNPs with targeting moieties such as transferrin, lactoferrin, or dopamine analogs further improves their delivery to the substantia nigra and striatum, key regions of dopaminergic degeneration in PD [101].

Despite the promise of these advanced delivery systems, several challenges remain, including the need for long-term safety data, scalable synthesis, and regulatory approval pathways. Potential immunogenicity, toxicity, nanoparticle aggregation, and interactions with the protein corona in biological fluids may affect biodistribution, efficacy, and safety.

## 4. Discussion

**Figure 4:**
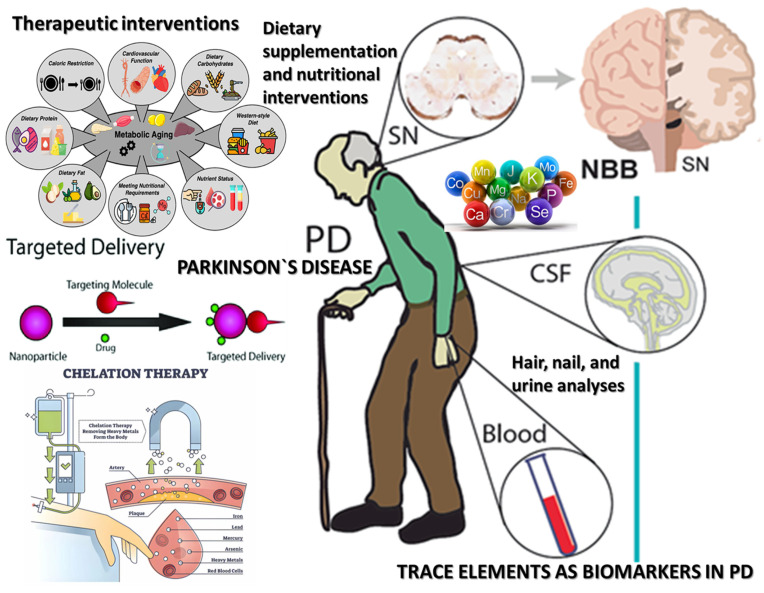
Integrative approaches to trace-element dysregulation and therapeutic strategies in PD. Interventions targeting metabolic aging, including caloric restriction, dietary macronutrient modulation (protein, fat, carbohydrates), cardiovascular function maintenance, and nutritional status optimization, are preventative strategies to mitigate trace-element imbalance and its sequelae. A multifactorial paradigm view combines early biomonitoring, dietary interventions, targeted drug delivery, and chelation therapy to address trace element-related pathophysiological mechanisms in PD.

The current synthesis regarding trace elements in PD (see Figure 4) reveals a complex and multifaceted relationship between metal homeostasis and neurodegeneration. A substantial body of literature supports the notion that disturbances in the metabolism of essential and toxic trace elements are closely linked to core pathological mechanisms in PD, including oxidative stress, mitochondrial dysfunction, α-synuclein aggregation, neuroinflammation, and ferroptosis. However, findings across studies vary considerably regarding directionality, magnitude, and clinical relevance, reflecting both methodological heterogeneity and the biological complexity of metal interactions in the human brain.

Variability in trace-element findings may arise from methodological factors, including differences in analytical techniques, sample type (serum vs. CSF vs. hair, nails vs. brain tissue), and pre-analytical conditions (storage, contamination, chelation status). Furthermore, inter-individual differences in age, sex, genetic background, diet, medication use (e.g., Levodopa), and comorbidities can substantially influence trace-element status.

Comorbidities and medications also exert substantial influence. Conditions such as anemia, diabetes, renal impairment, and inflammatory diseases affect trace elements’ metabolism and bioavailability [102]. For instance, chronic kidney disease can alter zinc and selenium levels [103], while liver dysfunction may impair copper metabolism [104]. Moreover, commonly prescribed medications in PD, such as Levodopa, anticholinergics, and monoamine oxidase-B (MAO-B) inhibitors [105], can impact gastrointestinal motility, nutrient absorption, and/or oxidative balance, indirectly affecting trace-element profiles. Polypharmacy and supplements’ use further complicate trace-element status, especially in older populations [51,106].

Lastly, genetic polymorphisms in metal transporters, antioxidant enzymes, and regulatory proteins (e.g., ferritin, transferrin, metallothioneins) contribute to inter-individual variability in metal handling and disease susceptibility [107]. These genetic differences are rarely assessed in human studies but are increasingly recognized as critical modifiers of trace-element effects [52].

Recent advances in metallomics can identify specific metal–protein complexes, oxidation states, or isotopic ratios that reflect unique biochemical processes in individual patients. Coupling metallomics with metabolomics, genomics, transcriptomics, and proteomics may reveal patient-specific regulatory networks involving metal transporters, chaperones, and metalloproteins, enabling more precise therapeutic targeting. Exploring trace elements in PD has yielded significant insights into their potential roles in pathogenesis, diagnostics, prevention, and therapeutic strategies [108]. However, substantial gaps in knowledge and translational application persist.

Recent meta-analyses support the involvement of essential trace elements, including iron, zinc, copper, manganese, and selenium, in Parkinson’s disease (PD). Specifically, circulating iron concentrations have been consistently observed to increase in the substantia nigra region of PD patients, as confirmed by quantitative susceptibility mapping (QSM) and susceptibility-weighted imaging (SWI) MRI techniques [109]. Conversely, meta-analyses evaluating peripheral fluids like serum, plasma, and CSF show inconsistent and generally nonsignificant differences in iron levels between PD patients and controls, suggesting compartment-specific discrepancies [109].

Regarding zinc, significant decreases in circulating serum and plasma zinc levels have been robustly documented through meta-analysis, indicating a potentially critical role for zinc deficiency in PD pathogenesis, possibly via impaired antioxidant capacity and altered enzymatic activities [110]. In contrast, findings from cerebrospinal fluid (CSF) zinc analyses remain inconclusive, likely due to smaller sample sizes [110].

Copper homeostasis also appears altered in PD, as evidenced by meta-analyses demonstrating elevated copper concentrations in CSF and reduced levels in peripheral compartments such as serum and plasma, suggesting copper dysregulation contributes to central nervous system toxicity, oxidative stress, and mitochondrial dysfunction in PD. Dietary copper intake has been inversely associated with PD risk, highlighting its potential neuroprotective effects when consumed in balanced amounts [47].

Finally, selenium levels in CSF have shown significant elevations among PD patients, with potential neurotoxic effects from chronic exposure, while peripheral levels exhibit heterogeneous outcomes [111].

The current literature is dominated by cross-sectional analyses, which do not allow for causal inference. Further longitudinal studies could clarify whether observed trace-element imbalances are antecedent risk factors, coincident pathophysiological events, or secondary responses to neurodegeneration. Randomized controlled trials targeting specific mechanisms, such as ferroptosis inhibition via selenium supplementation, or α-synuclein aggregation suppression through copper or iron chelation, are critically needed, focusing on biomarker-guided patient stratification and multi-domain outcome measures.

Another priority involves stratifying PD patients by clinical subtypes (e.g., tremor-dominant vs. postural instability), disease duration, and relevant genetic polymorphisms affecting metal transport and metabolism. This would facilitate precision-medicine approaches and avoid therapeutic generalizations that overlook inter-individual variability in trace-element dynamics.

We also recognize the importance of environmental exposure mapping in future studies. Regional variability in soil composition, water mineral content, and dietary customs can profoundly affect trace-element homeostasis, yet these factors are rarely accounted for in existing PD research. Geospatial analysis combined with exposure monitoring and dietary assessment tools could help delineate modifiable risk factors and support preventive strategies at the population level.

We highlight the need to incorporate emerging technologies into trace-element research in parallel with these epidemiological and interventional priorities. High-resolution ICP-MS, synchrotron-based X-ray fluorescence imaging, and advanced MRI techniques such as quantitative susceptibility mapping (QSM) provide unparalleled sensitivity and spatial resolution for assessing metal distribution in vivo and ex vivo. These tools allow for the quantification and localization of metals in neuroanatomically relevant regions, such as the substantia nigra, with potential applications in diagnosis, monitoring, and therapeutic evaluation.

The principal reason meta-analysis was deemed infeasible stems from the substantial methodological heterogeneity observed across the included studies. Differences existed in nearly every key domain—study design (e.g., case–control, cohort, experimental models), trace-element measurement techniques (e.g., ICP-MS, AAS, ELISA), biological matrices analyzed (serum, plasma, CSF, hair, postmortem brain tissue), units of measurement, and statistical treatment of data (e.g., raw concentrations, adjusted means, or transformed variables). Moreover, the reported outcomes were rarely harmonized; some studies focused on absolute trace-element levels, while others reported elemental ratios, redox indices, or indirect biomarkers such as metalloprotein expression. This diversity precluded the generation of standardized effect sizes or the pooling of data under a fixed- or random-effects model without introducing profound bias or violating assumptions of statistical homogeneity.

In addition, studies differed markedly in terms of participant characteristics, including disease duration, severity (Hoehn and Yahr stage, UPDRS scores), age distribution, geographic region, comorbid conditions, and exposure history. Few studies applied uniform stratification or adjusted for key covariates such as medication use, dietary intake, renal or hepatic function, or systemic inflammatory status, all of which influence trace-element bioavailability and distribution. The absence of individual participant data (IPD) further limited our ability to perform subgroup analyses or apply meta-regression to explore potential moderators of effect.

Despite these limitations, future reviews in this area may benefit from more refined quantitative methods. One promising avenue is the use of *network meta-analysis* or *Bayesian hierarchical modeling* to integrate studies with differing designs and reporting metrics, provided that standardization and imputation strategies are implemented. Another approach could involve *individual patient data meta-analysis*, which, although logistically demanding, would allow for the direct adjustment of confounders and an exploration of within-study heterogeneity. Additionally, the application of *machine learning clustering techniques* to pooled biomarker datasets may reveal underlying data patterns and stratify patient subgroups based on trace-element profiles.

Finally, we recommend the establishment of international data harmonization initiatives and minimal reporting standards for trace-element studies in neurodegenerative diseases. Such efforts would facilitate future meta-analyses by promoting consistency in outcome measures, analytical protocols, and data transparency. Until such standardization is achieved, we maintain that qualitative synthesis remains the most rigorous and ethically defensible means of integrating the current heterogeneous body of literature.

## 5. Conclusions

The accumulating body of evidence—from postmortem analyses, biofluid and tissue profiling, neuroimaging, animal models, and cell-based assays—strongly supports the involvement of trace-element imbalance in the initiation and progression of PD. Advances in analytical technologies and multi-omics platforms allow for a detailed characterization of metal species, binding partners, and their dynamic interactions with cellular systems. Such insights reveal trace elements not only as passive correlates of pathology but also as active modulators of critical disease pathways, including α-synuclein aggregation, mitochondrial failure, and chronic glial activation. These discoveries open novel opportunities for the development of early diagnostic biomarkers, risk stratification tools, and targeted interventions, including personalized nutritional supplementation, metal-specific chelators, and (possibly) nanoparticle-based delivery systems.
